# The role of arthroscopic release of gluteal muscle contracture in improving patellofemoral instability

**DOI:** 10.1186/s13018-019-1187-9

**Published:** 2019-05-28

**Authors:** Jing Biao Huang, Heng’an Ge, Ying Lei Zhang, Cen Tao Liu, Chao Xue, Yi Chao Chen, Peng Wu, Biao Cheng

**Affiliations:** Department of Orthopedics, Shanghai Tenth People’s Hospital, Tongji University, School of Medicine, 301 Yanchang Middle Road, Shanghai, 200072 China

**Keywords:** GMC, Arthroscopy, Patellofemoral instability, CT

## Abstract

**Background:**

Gluteal muscle contracture (GMC) is a notable problem in some developing countries and includes features such as a snapping sound of the hip, abnormal gait, and unusual posture when patients squat with the knees together. Arthroscopic release can not only resolve symptoms, as previously reported, but can also greatly improve accompanying patellofemoral instability. This study was conducted to evaluate the effect of arthroscopic release of GMC on patellofemoral instability and its underlying mechanism.

**Methods:**

A total of nearly 500 patients who underwent arthroscopic release of GMC over 2.5 years were filtered, and 54 patients were enrolled in the study. The selected research subjects all had combined patellofemoral instability preoperatively. The Lysholm scores and CT scans of the knee were evaluated pre- and postoperatively.

**Results:**

The mean follow-up time was 12.2 months. All of the surveyed patients had satisfactory clinical outcomes for hip snapping sounds and abnormal gait. In addition, a significant difference (*p* < 0.05) was observed between pre- and postoperative Lysholm scores, along with significant knee pain relief. Furthermore, the changes in CT scan parameters were significant as well. The average patellar tilt angle (PTA), patellofemoral index (PFI), and lateral patellar displacement (LPD) were obviously decreased (*p* < 0.05) after the release. Conversely, the mean lateral patellofemoral angle (LPFA) showed a clear difference (*p* < 0.05) between preoperative and postoperative CT examinations.

**Conclusions:**

Arthroscopic release of GMC can reduce the tilt and lateral shift of the patella and enhance its stability due to the release of the iliotibial band.

## Background

Gluteal muscle contracture (GMC) is a disease that mainly occurs in adolescents and infants, and the majority of these patients receive repetitive buttock injections at very young ages [1]. GMC was first identified by Valderrama, a British doctor [2]. However, in China, this phenomenon is rather ubiquitous, with an incidence rate ranging from 1 to 2.5% [3]. Due to the contracture and fibrous degeneration of gluteal muscle, patients present with snapping sounds of the hip, an abnormal gait, and unusual posture when they squat with the knees together [4]. The onset of GMC is usually bilateral, and boys have a higher incidence rate than girls. Indisputably, surgery is the first treatment choice, and arthroscopic release is the gold standard globally, as first proposed by Wentao Zhang in 2002 [5]. Arthroscopic release of GMC has the benefits of being minimally invasive, having a wider surgical field, fewer complications, rapid recovery, and greater satisfaction, compared to open surgery [6–9].

**Fig. 1:**
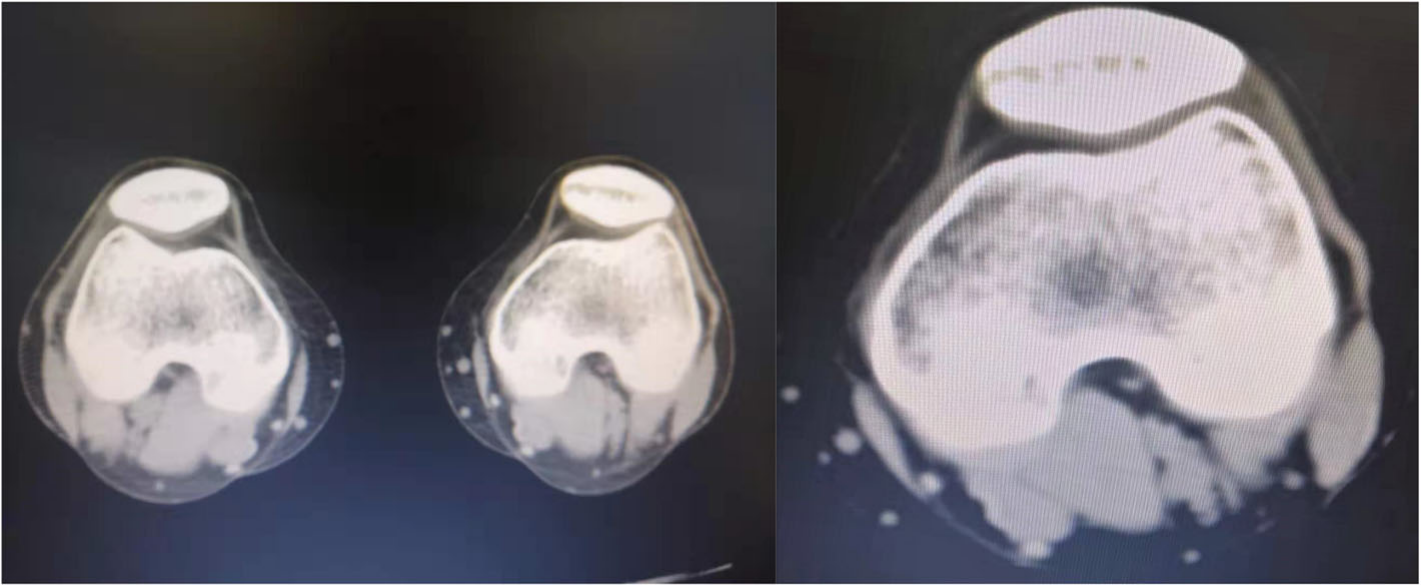
A 22-year-old woman with bilateral knee pain (bilateral CT images) for more than 10 years. Postoperative CT images show significant improvement

Furthermore, concomitant symptoms, including pelvic tilts, lumbar muscle strain, and patellofemoral instability, can occur in patients with severe contractures and longer medical histories. Many patients suffer from knee pain, especially when crouching and walking up and down stairs, without any preceding trauma of the knee. Their symptoms are mainly induced by GMC, as reported by Zhao et al. [10] According to our clinical experience, the associated patellofemoral instability can be relieved to some extent after endoscopic release, with some patients showing dramatic improvement postoperatively (Fig. 1). However, few studies have examined the relationship between gluteal muscle release and improvement of patellofemoral instability. This study was undertaken to examine this relationship and to provide better solutions for these patients.

## Materials and methods

### Subjects

The chief physician selected the study subjects among patients who were diagnosed with GMC and underwent arthroscopic release from January 2016 to June 2018 in the Tenth People’s Hospital of Shanghai. For patients with concomitant knee pain, pelvic radiographs and CT scans of the knee were performed to identify patellofemoral instability. The subjects included 24 male and 30 female patients with ages ranging from 16 to 37 years and a mean age of 26.5 ± 4.4 years. All patients underwent CT scans pre- and postoperatively, which showed severely affected knees with bilateral GMC. Regarding the clinical manifestations, the subjects all had obvious GMC symptoms, with girdle-like contracture of the iliotibial band, along with knee pain, weak knees upon standing, and varying degrees of shifting of the patella outward when squatting. The exclusion criteria were as follows:

1. Patients with meniscus injury and/or cruciate ligament injury
2. Emergent patella fracture
3. Other diseases that could lead to knee pain, for example, rheumatoid arthritis
4. Patients who underwent muscular release more than once
5. Pelvic radiographs revealing the existence of leg length discrepancy and an asymmetric pelvis
6. Other factors that resulted in patellofemoral instability, for example, trochlear dysplasia, lateralization of the tibial tubercle, pes planus, and absence of the medial patellofemoral ligament
7. Other factors that could result in snapping hip, for example, intra-articular snapping

### Surgical procedure and rehabilitation

All patients underwent bilateral release of the gluteal muscles and definitive physiotherapy. Typically, each patient was placed in the lateral position on the operating table after induction of general anesthesia. By moving the hip joint repeatedly, the snapping area was targeted, and incisions were marked before sterilization. Then, the superficial fascia and contracture tissues were incised. The first step involved the forward release of the tensor fasciae latae and superficial fascia. The second step was to release the contracture tissues along the spinal iliac edge and to release the metamorphic gluteus medius and gluteus minimus if necessary. Finally, an arthroscopic probe was used to examine the area near the greater trochanter to remove any remaining tissues exhibiting contracture. The iliotibial band was the main contracture part and was the principal release tissue in most cases. Care had to be taken in releasing the tissues to avoid damaging the sciatic nerve. After the arthroscopic probe confirmed no remaining contracture of tissues and movement of the hip joint with no snapping due to obstacles, the incision was closed, and the wounds were debrided.

Drainage is generally not recommended to allow early exercise. Patients were encouraged to bear full weight on the second day after surgery with the help of others. During sleep, the two legs were bound with a restraint strap for at least one week. The patients were allowed to flex the hip and knee and cross the legs side by side. Embracing the legs with the knees together as close to the chest as possible (passively or positively) was also repeated three to five times.

### Data collection and analysis

**Fig. 2:**
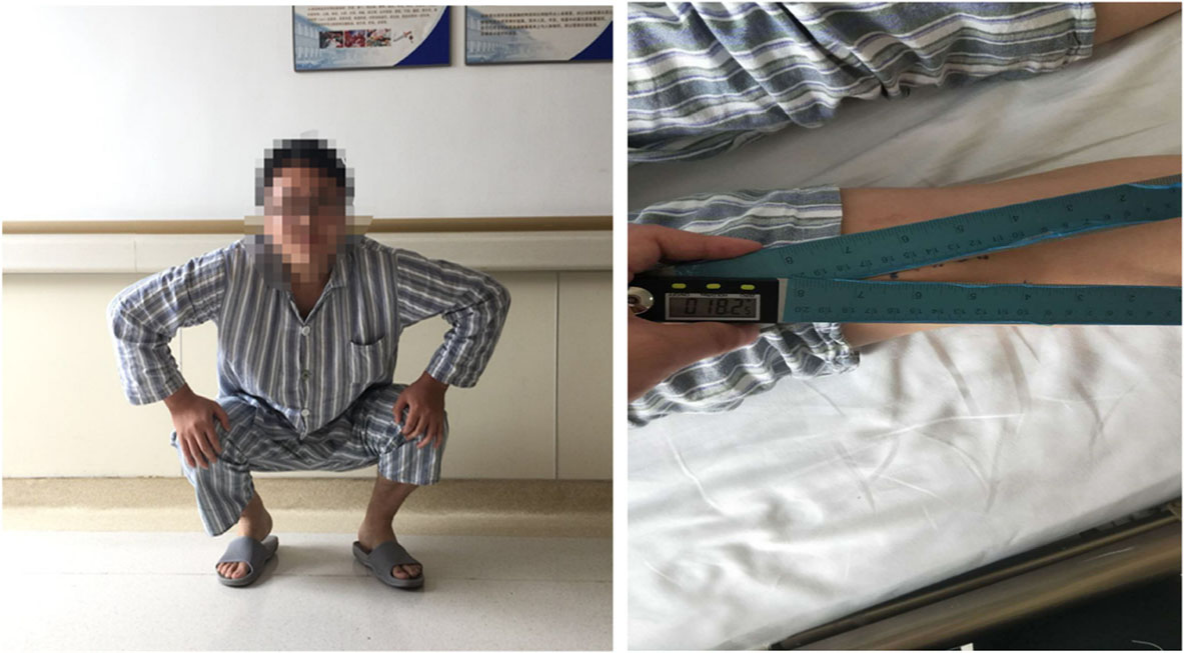
A patient with GMC and knee pain and measurement of the *Q* angle

We measured the quadriceps angle (*Q* angle) (Fig. 2) and assessed the Lysholm knee scores both before and after the operation. CT examinations of the ipsilateral knee were also conducted twice to compare patellofemoral instability. Values on CT images including the patellar tilt angle (PTA), patellofemoral index (PFI), lateral patellar displacement (LPD), and lateral patellofemoral angle (LPFA) were used to evaluate the improvement of patellofemoral instability. All values were independently measured by two researchers, and their average was calculated. If obvious differences were present between the measured results, the reasons for the discrepancy were analyzed, and the values were remeasured in cases of measurement errors.

### Statistical analysis

Statistical analysis was conducted using SPSS software, version 19. Paired *t* tests were used to compare the pre- and postoperative values. A *p* value less than 0.05 was considered to indicate a significant difference.

## Results

In total, 52 patients were monitored, resulting in a follow-up rate of 96.3%. The mean follow-up time was 12.2 months. All of the surveyed patients (52 patients) had satisfactory clinical outcomes regarding snapping sounds and abnormal gait after regular exercise. There were no cases of blood vessel damage, nerve damage, or wound infection. The mean *Q* angle before and after the operation greatly decreased from 19.7 ± 2.1 to 15.8 ± 1.5 (*p* < 0.05), indicating that the outward component force of the patella had decreased. Concerning knee symptoms, 50 patients gained significant relief of knee pain, while two patients had dissatisfactory improvement in knee pain. Neither their symptoms nor CT images indicated postoperative improvement, which indicated that their patellofemoral instability was due to other reasons. The average Lysholm scores increased significantly from 58.3 ± 9.9 to 76.9 ± 5.7 (*p* < 0.05) after the surgery. Moreover, the changes in CT scan parameters showed significant differences (*p* < 0.05) as well. The average PTA, PFI, and LPD markedly decreased (*p* < 0.05) after release. Conversely, the mean LPFA showed a clear increase (*p* < 0.05) between the preoperative and postoperative CT examinations (Table 1). These change in CT values revealed a salient increase in patellofemoral joint stability.

**Table 1:** Preoperative and postoperative values (last follow-up)

Value	Preoperative	Postoperative
*Q* angle (°)	19.7 ± 2.1	15.8 ± 1.5
PFI	1.87 ± 0.16	1.49 ± 0.92
LPFA (°)	− 0.60 ± 1.01	6.53 ± 0.67
PTA (°)	22.8 ± 2.5	12.9 ± 2.8
LPD (cm)	0.52 ± 0.19	0.22 ± 0.19

## Discussion

The pathogenesis of GMC is complex and has not yet been fully determined. A relatively broad consensus has resulted in most patients receiving repeated buttock injections. Additionally, GMC is more common in China than in other countries due to the frequent use of benzyl alcohol as a diluent for intramuscular injection of penicillin since 1970 [9, 11]. Furthermore, the age at injection, injection frequency, and interval between injections have been associated with the likelihood of disease. Genetic factors also play an important role. In 2015, Zhang et al. described a mechanism that regulates GMC that consists of the TGF-β/Smad pathway and its downstream effectors [12]. Trauma and infection of the hip are other rare etiological factors.

Currently, arthroscopic release is the gold standard for the treatment of GMC. Many studies have been conducted on the selection of the incision site, comparison with open surgery, and classification, but few studies have focused on the accompanying symptoms. Per the authors’ experience, anterior knee pain caused by patellofemoral instability is a major concomitant symptom in these patients, especially patients older than 30 years old or those with severe GMC. Other studies have also revealed that GMC can lead to knee pain and have confirmed that patellofemoral instability is strongly correlated with iliotibial band contracture [10, 13]. Nevertheless, there is no direct evidence indicating that the release of GMC can improve patellofemoral instability. Therefore, we conducted this study and used CT scans to provide an innovative perspective in assessing the improvement of patellofemoral instability.

Regarding the assessment of patellofemoral instability and knee pain, we eventually selected several quantitative indices for pre- and postoperative measurement. The *Q* angle is an indicator that is determined by the patella tendons and quadriceps and reflects the vector force of the outward shift or ingression [14]. The Lysholm knee score is an internationally accepted standard for estimation of the knee joint, introduced by Lysholm in 1982, with great sensitivity, reliability, and validity. We measured three indices to assess the patellar tilt using CT images. The LPFA indicates an abnormal tilt of the patella if the value is − 8° or if only a medial opening is present [15]. In our study, a medial opening of the LPFA was recorded as negative. The PFI is the ratio of the narrowest width of the inside and outside patellofemoral joints, and a normal value is − 1.6. The PTA is formed by a line parallel with the lateral patellar facet and a second line parallel to the posterior condylar line [16]. This study also used an indicator of LPD, which reflects the outward shift of the patella. Additionally, the tibial tubercle-trochlear groove (TT-TG) does not change because we do not address the tubercle of the tibia or the trochlear groove, as confirmed by CT comparisons.

**Fig. 3:**
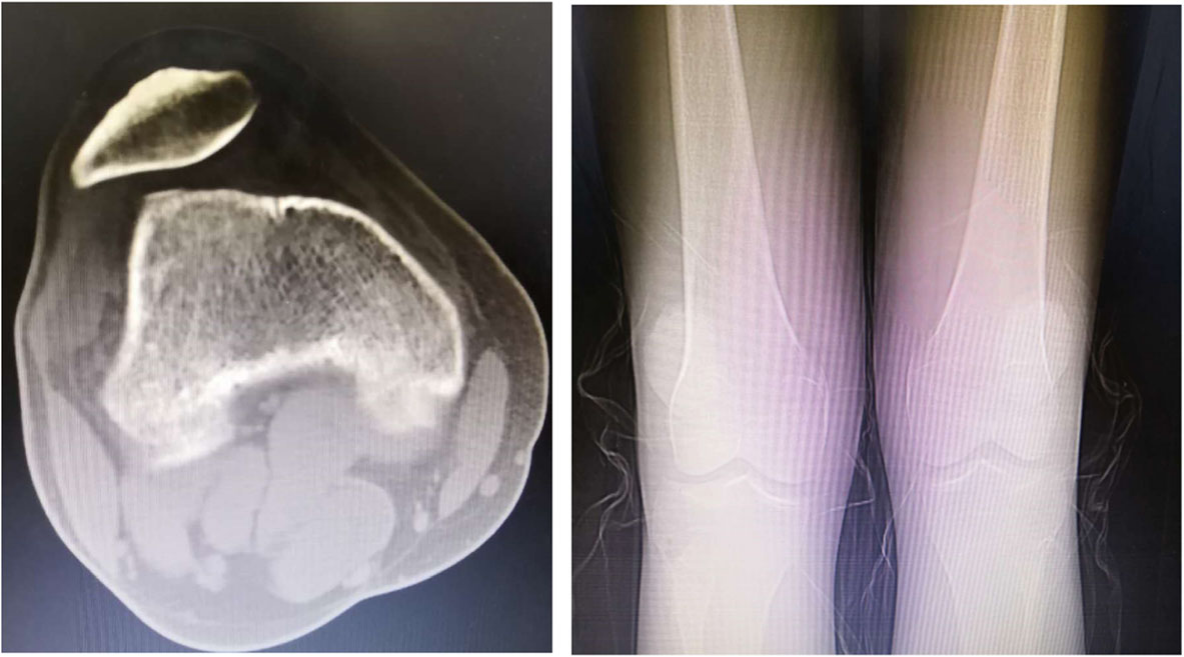
Preoperative CT and X-ray images show patellofemoral instability and femoral trochlear dysplasia

Many injuries are associated with anterior knee pain and patellofemoral instability. Common contributors, such as meniscus injury, ligamentous injury, osteochondral injury, and patella fracture, can be precisely diagnosed through medical history, clinical symptoms, and imagological examination. Using these methods, we excluded these patients from our sample, particularly those with a trauma history. In addition, an abnormal anatomical position and track of the patella are also probable causes. Most knee pain associated with GMC is due to these factors. Contracture of the iliotibial band, tightness of the lateral retinaculum and an increased *Q* angle are main anatomical factors that predispose individuals to patellofemoral instability. GMC patients experience strain of the lateral patellar retinaculum caused by contracture of the gluteal muscles, femoral fascia, and most definitively the iliotibial band. Lateral retinaculum strain resulted in the limited inward motion of the patella and excessive lateral pressure syndrome (ELPS), a disease characterized by acute anterior knee pain when patients squat and stand [10]. Hypertension of the lateral retinaculum also led to patellar tilt and outward shift, which in serious cases could transform into subluxation or luxation. In addition, the iliotibial band contracture and compensatory, increased strength of the hip abductors contributes to the abduction of the lower limbs and enlargement of the *Q* angle, which increases the outward tensile stress of the patella. The shape of the patella will also change if the patient is not treated in a timely manner. Compared to individuals without this condition, GMC patients tend to have thicker patellar subchondral bones of the lateral facet, lateral osteophytes of the patella, and a lateral patella that bestrides the lateral femur condylar. These phenomena reveal the ever-quickening degeneration of the patellar joint among GMC patients. Furthermore, the static stability of the patella was maintained by the balance of the medial and lateral retinaculum and by the femoral trochlea. When ELPS occurs, this important balance is disrupted, making the patella more likely to tilt and shift to the side. On some patients’ CT images, femoral trochlear dysplasia was also present (Fig. 3), which also greatly affected the static stability of the patella and patellar tracking, but these cases were excluded in the study. In all, GMC can undoubtedly cause knee pain and patellofemoral instability, and it has become the most common pathogenesis of patellofemoral instability and related patellofemoral disease in juveniles.

GMC can also result in the enhancement of gluteal muscular strength, but it also causes a diminution of muscle tone, which decreases the length of the gluteus maximus. The iliotibial band starts at the tensor fasciae latae on the iliac crest and then is connected to the top and upper rear of the gluteus maximus. Thus, the starting point of the iliotibial band would be stretched if the length of the gluteus maximus was decreased. In addition, the iliotibial band extends across the lateral patellar retinaculum and finally stops at the lateral femoral condyle. This traction stimulates hypertension of the lateral retinaculum and increases the outward tensile stress of the patella. Contraction and traction of the iliotibial band also cause intrinsic muscle strength enhancement, which exerts an outward and upward tensile stress on the patella and thus increases the instability of the patella. Thus, the contracture of the iliotibial band is the main factor that determines the instability of the patellofemoral joint and it is the main release object during the operation.

**Fig. 4:**
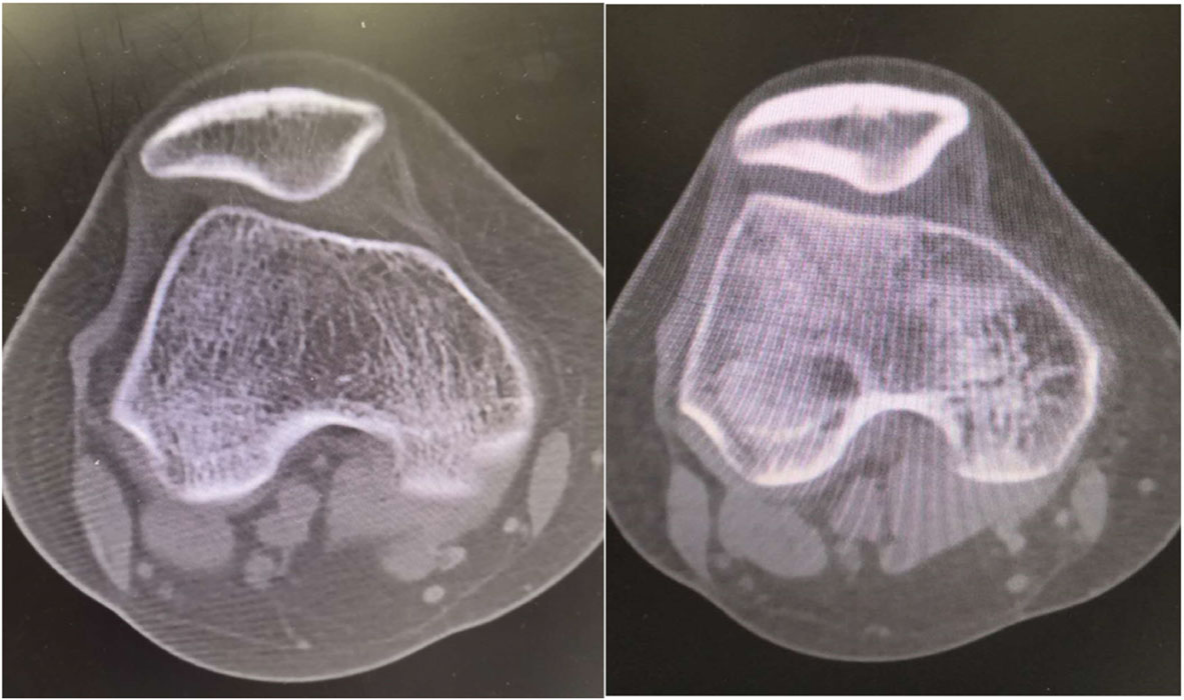
Preoperative CT images of a 27-year-old patient (left) and ipsilateral images on the second day after surgery (right)

According to our experience, the arthroscopic release of the gluteus muscle and soft tissues (especially the iliotibial band) can result in significant improvement of patellar instability. A study of 52 cases even showed that GMC release could relieve knee osteoarthritis [13]. These variations in the patellofemoral joint are visible by pre- and postoperative CT comparisons. Some patients had sufficient relief on the second day after the operation (Fig. 4), which strongly confirmed that this change was due to correct and timely release. Several typical cases could reveal the relationship between iliotibial band release and patellofemoral joint stability in this study. One of them was a 27-year-old female with a snapping sound of the hip, an abnormal gait, and severe pain of the right knee. A physical examination confirmed that the main muscle exhibiting contracture was the iliotibial band, which had a large range and produced obvious snapping. The patellar tilt test was positive, and the patellar apprehension sign was negative. Other diseases that could lead to anterior knee pain such as meniscus injury and ligamentous injury were excluded. A preoperative CT examination showed malalignment of the patellofemoral joint (Fig. 4). We performed a thorough search and released the iliotibial band and surrounding soft tissues during the operation. The patient reported relief of knee pain on the second day after surgery. Therefore, we re-examined the right knee on the second day after surgery and discovered a significant improvement of the tilt and outward shift of the patella on CT images. After 6 months of follow-up, the patient showed a normal gait and posture and thorough relief of knee pain. The patient experienced a rapid and obvious improvement of patellofemoral joint stability after we released the iliotibial band, and this was verified by postoperative clinical symptoms and CT examinations. This case also demonstrated that patellofemoral instability associated with GMC is mostly related to iliotibial band contracture, and full arthroscopic release of the tense iliotibial band could improve this instability. Therefore, the most important cause of symptom relief is the release of contracture of the iliotibial band, which is also the most fundamental pathogenesis of GMC. The tension of the lateral patellar retinaculum and abnormal *Q* angle are also decreased when the iliotibial band is released. As noted previously, the outward tensile stress on the patella is markedly decreased through multiple effect. Finally, arthroscopic technology provides a wider scope, exhibits better organization, and results in a more thorough release of contraction than open surgery. It is also the first choice for patients with GMC and patellar instability.

The main limitation of this study is that we did not conduct arthroscopic exploration of the knee to examine the condition of the cartilage. Additionally, the sample size was small, and the long-term effects need to be observed in these patients.

## Conclusions

1. Arthroscopic release of GMC can reduce the tilt and lateral shift of the patella and enhance its stability via the release of the iliotibial band.
2. To prevent and reduce patellar instability, the tissues exhibiting contracture should be released as early as possible. Early treatment results in better outcomes and slower degeneration of the patellofemoral joint.

## Abbreviations

ELPS: Excessive lateral pressure syndrome
GMC: Gluteal muscle contracture
LPD: Lateral patellar displacement
LPFA: Lateral patellofemoral angle
PFI: Patellofemoral index
PTA: Patellar tilt angle
TT-TG: Tibial tubercle-trochlear groove
